# Mechanical and Hydrothermal Aging Behaviour of Polyhydroxybutyrate-Co-Valerate (PHBV) Composites Reinforced by Natural Fibres

**DOI:** 10.3390/molecules24193538

**Published:** 2019-09-30

**Authors:** Karolina Mazur, Stanisław Kuciel

**Affiliations:** Faculty of Mechanical Engineering, Cracow University of Technology, ul. Warszawska 24, 31-155 Cracow, Poland; stask@mech.pk.edu.pl

**Keywords:** PHBV, wood fibers, basalt fibers, injection molding, biocomposites

## Abstract

Biodegradable composites based on poly (3-hydroxybutyrate-co-3-hydroxyvalerate), reinforced with 7.5% or 15% by weight of wood fibers (WF) or basalt fibers (BF) were fabricated by injection molding. BF reinforced composites showed improvement in all properties, whereas WF composites showed an increase in Young’s modulus values, but a drop in strength and impact properties. When compared with the unmodified polymer, composites with 15% by weight of BF showed an increase of 74% in Young’s modulus and 41% in impact strength. Furthermore, the experimentally measured values of Young’s modulus were compared with values obtained in various theoretical micromechanical models. The Haplin-Kardas model was found to be in near approximation to the experimental data. The morphological aspect of the biocomposites was studied using scanning electron microscopy to obtain the distribution and interfacial adhesion of the fibers. Additionally, biodegradation tests of the biocomposites were performed in saline solution at 40 °C by studying the weight loss and mechanical properties. It was observed that the presence of fibers affects the rate of water absorption and the highest rate was seen for composites with 15% by weight of WF. This is dependent on the nature of the fiber. After both the first and second weeks mechanical properties decreased slightly about 10%.

## 1. Introduction

A growing issue has emerged recently due to the increase in the production of non-degradable, petrochemical polymer composites. These materials not only have good mechanical properties but are also inexpensive and easy to produce. However, after their period of use, they typically cannot be recycled and cause environmental pollution. Therefore, bio-based and biodegradable composites have been the subject of extensive research. Bio-based polymers can be synthesized from petrochemical raw materials or bio-resources. Among the latter group, which has attracted increasing interest from the scientific community and industry, the most popular are polylactide and polyhydroxyalkanoate (PHA) polymers. The main advantage of these polymers is their biodegradability. PHAs are aliphatic polyesters produced by micro-organisms that carry out fermentation to accumulate a carbon source and energy storage material. One of the most popular polymers in this group is poly (3-hydroxybutyrate-co-3-hydroxyvalerate) (PHBV) [1,2]. It is a copolymer of poly (3-hydroxybutyrate) and hydroxyvalerate (HV). Physical and mechanical properties of PHBV are similar to those of conventional thermoplastics such as polyethylene and polypropylene, but it is biodegradable and biocompatible, and one can easily change its properties by manipulating the content of HV. Low content of HV in PHBV leads to reduction of flexibility, elongation and impact strength, and higher content decreases its brittleness due to inhibition of secondary crystallization [3]. PHBV-based composites are becoming increasingly the object of scientific interest [4,5].

The above mentioned advantages make it a green alternative to the former compounds. However, factors such as complexity and high cost of production compared to petroleum-based composites, slow crystallization rate and low impact toughness still limit the application of PHBV [6]. Therefore, reinforcing it with fillers is a potential solution to the previously mentioned issues and a field of study. In the case of biopolymers, it is reasonable to use natural fillers that will make the produced composites environmental-friendly and biodegradable. A great interest in fiber-reinforced polymer composites has been observed in recent years. These composites exhibit very good mechanical properties, in particular, high stiffness and strength. Additionally, they have low weight and are inexpensive. The high strength-to-weight ratio of natural fibers makes them an excellent alternative to synthetic fibers. Besides, natural fibers have good insulating properties, and at the same time are easy to recycle and are biodegradable. PHBV has recently been studied as a matrix in green composites with WF, cellulose, bamboo, flax, kenaf, and others [7,8,9]. As can be seen, the most common natural fillers are fibers comprising lignocellulose. Studies on lignocellulose fillers showed that tensile, flexural and impact strength were not improved, but Young and flexural modulus increased. These observations were confirmed by Srubar et al. [10]. Researchers studied the effect of oak wood flour (OWF) on the properties of PHBV-based composites, with fiber additions ranging from 0 to 46 vol%. They noticed that the addition of fibers along with their increased in content increased Young’s modulus from 3.80 GPa to 4.32 GPa (36 vol%). However, as the OWF content increased, the tensile strength values systematically decreased from 34.8 MPa to 21.4 MPa (36 vol%) and the ultimate strain from 1.6% to 0.8%. Another group, Hassaini et al. [11] showed similar relationships. The study PHBV composites with 10, 20 and wt% of olive husk flour (OHF). Researchers noticed that with the increasing content of fibers, composites becomes stiffer, due to the rigid nature of the lignocellulosic fibers and Young modulus increased from 3.14 GPa to 5.43 GPa. However, both stress and elongation at break decreased, due to the weak interaction between matrix and fibers. Addition of fibers caused an increase in water absorption from 0.051% to 0.423% (30% of OHF). This increase occurred due to the introduction hydrophobic nature of fibers. It can be assumed that lignocellulosic fibers could give higher effects, after increasing the adhesion matrix/fibers e.g.: by surface treatment of the fibers or addition of nucleating agent.

Basalt fibers (BF) are becoming an increasingly common natural reinforcing material. Basalt is an igneous rock which can be melted and drawn into continuous filaments using processing techniques similar in many respects to that used to make glass fibers (GF). BFs have better mechanical properties than GFs, specifically tensile modulus (BF: 90 GPa and GF: 76 GPa) [12]. Due to their high resistance, thermal strength and chemical resistance, BFs can replace carbon fibers (CF), if composites do not require as high a strength as CF composites. Therefore, BF bridge the performance gap between GF and CF. BF appear to be perfect fillers of natural origin for biodegradable matrices for improving mechanical properties. Studies on composites based on PHBV reinforced with BF are very rarely described in the literature. More often it can be found information about PLA biodegradable polymers reinforced with BF [13]. BF-reinforced composites show an increase in mechanical, thermal and processing properties [13]. Ying et al. [14] tested PLA/BF composites up to 20 wt% content of fibers. Composites with 20 wt% of BF improved about 30% and 47% of strength and tensile modulus, respectively. The addition of BF reduced cold crystallization temperatures (T_cc_) from 116.1 °C to 112.2 °C, which confirms the act of BF as a nucleating agent.

This paper describes the manufacturing and evaluation of the properties of biodegradable PHBV-based biocomposites with various amounts of fibers of different origins (WF and BF). This research involved comparing the nature of strengthening of PHBV composites with fibers of various origins. Whereas, composites with lignocellulose fillers were previously tested, composites based on PHBV from BF have not been studied thus far. The composites were subjected to physical and mechanical tests at three different temperatures (−24 °C, +23 ± 2 °C, and +80 °C) and to morphological analysis to assess the reinforcement. The effect of hydrothermal aging on water absorption and mechanical properties was investigated. Furthermore, the experimental results obtained in this study and the theoretical results calculated using various theoretical micromechanical models of Young’s modulus were compared.

## 2. Results and Discussion

### 2.1. Fractographic Analysis

**Figure 1:**
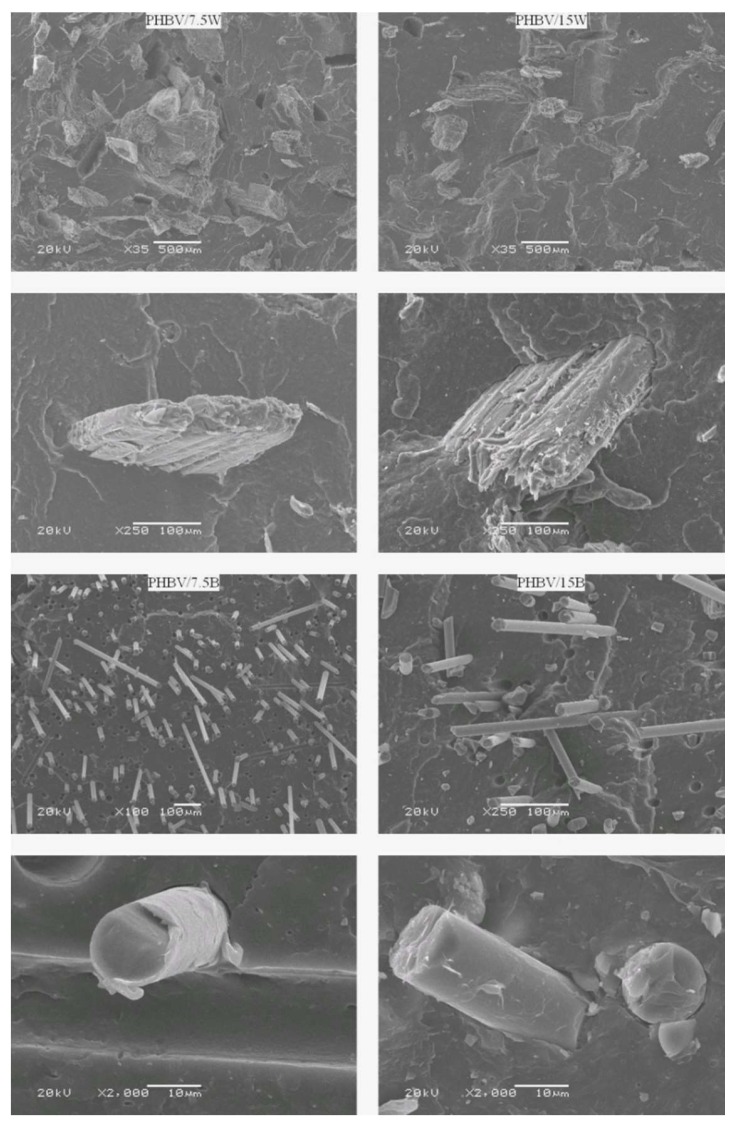
SEM micrographs of poly (3-hydroxybutyrate-co-3-hydroxyvalerate) (PHBV) composites with wood fibers (WF) and basalt fibers (BF).

Figure 1 shows the fractures in the surface of the samples of PHBV composites with BF and WF after the tensile test. The surfaces of the composites with BF and WF exhibit brittle fracture. As can be seen from the SEM pictures, both BF and WF were evenly distributed in the matrix without significant agglomeration. BF were partially coated with a polymer. However, black rings around BF were also visible, testifying to the lack of good fiber/matrix adhesion and contributing to the formation of local deformations during the tensile test. The images show that the fibers, though pulled out of the matrix (pull-out phenomenon), did not break along with the fracture surface. The pull-out phenomenon was much less common in WF, where a significant part of the wood fibrils was coated with a polymer. This is mainly related to the structure of WF consisting of individual microfibrils—they form an uneven fiber surface, consequently increasing the area of fiber/matrix interaction. There was poor adhesion between the fiber and a decrease in strength for composites with WF, and only a slight increase in strength for BF composites [15]. In the case of BF improved in properties, it was connected with BF high mechanical properties. Compatibilizer additives or special surface treatment may improve fiber/matrix adhesion in the tested composites, and thereby significantly improve the mechanical properties of composites for both the BF and WF. Although in WF composites the pull-out phenomenon was less prominent, one should not forget that WF, as compared to BF, are simply too short to induce toughening mechanisms hindering the brittle fracture.

### 2.2. Coefficient of Thermal Expansion (CTE)

The dimensional stability of polymer composites is a very important feature, especially for materials used for engineering purposes. A low CTE value is a desirable feature and extends the area of application of polymer materials. The positive influence of natural fillers on the dimensional stability of composites has been widely described in the literature [16]. Both WF, which contains cellulose with high thermal expansion coefficient, and BF with high thermal properties, improved CTE [17,18]. Table 1 presents CTE calculations in the temperature range from 30 °C to 70 °C. The lowest results were obtained for composites with BF (PHBV/15B: 42.33 × 10^−6^/°C), and for all composites it has been observed that as the fiber content increases, CTE decreases. The main reason for the reduced CTE is the nucleating effect of natural fibers [19]. Natural fibers initiate and accelerate the formation of polymer crystals which restrict polymer chain movements to very low levels. Similar observations were made by Singh and Mohanty who studied the properties of PHBV-based composites reinforced with WF [20]. As the fiber content increased, the value of CTE decreased from 182 × 10^−6^/°C (neat PHBV) to 173 (30% by weight) and 154 (40% by weight) ×10^−6^/°C. It is worth noting that in the case of the presented research, the CTE decreased for composites with 15 wt% of WFs was 26%, and in the cited work only by about 5% for composites with 30 wt% of fibers.

### 2.3. Mechanical Tests

**Figure 2:**
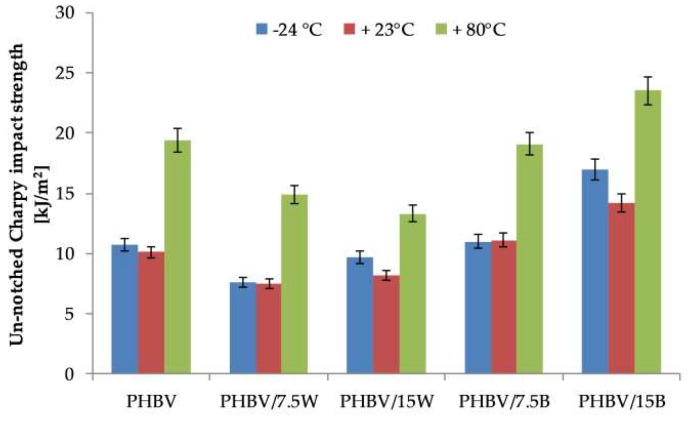
Un-notched Charpy impact strength of PHBV and its composites at −24, 23 and 80 °C.

Figure 2 shows the results of Charpy impact test at various temperatures (−24 °C, +23 ± 2 °C and +80 °C). The highest values of impact strength were recorded at elevated temperatures. This was related to the increase in the value of elongation at break compared to the values obtained at other temperatures. The addition of WF caused a slight decrease in impact strength. This was caused by an increased presence of a polar group of WF compared to neat PHBV, possibly leading to a poor adhesion of particles. Additionally, an increase in fiber content increases the possibility of agglomeration of WF, which leads to the formation of potential stress concentration zones [21]. This was confirmed by other scientists who studied WF composites [22,23]. Altun et al. [24] conducted research on PLA green composites reinforced with wood flour (WFL). The addition of WFL caused a significant reduction in the value of impact strength, regardless of its amount or surface treatment. This decrease was about 50% compared to neat PLA. PLA-based composites with 30% by weight of WFL recorded 5.2 ± 0.3 kJ/m^2^, composites with alkaline treatment recorded 6.1 ± 0.9 kJ/m^2^, while the unmodified polymer recorded 10.0 ± 1.6 kJ/m^2^. In our study, this decrease was only about 20% in the case of WF.

However, the BF additive improved the impact strength of the materials at all three temperatures. This was related to the high impact strength values of BF. BF reinforcement increased the absorption of impact energy and improved the fracture propagation of components. The pull-out phenomenon observed in SEM photos caused the extracted fibers to provide significant energy dissipation in the impact test. Also, the lack of plastic deformation at the breakthrough zones suggested that the extracted fibers caused additional dissipation of energy and the creation of new surfaces. Therefore, despite the higher strain at break for neat polymer (1.9%) than for PHBV/15B (1.2%), composites with BF have higher impact strength values by about 20%, and PHBV/7.5B values remain at the same level as neat PHBV, at 19.1 kJ/m^2^ and 19.4 kJ/m^2^ at elevated temperatures, respectively.

**Figure 3:**
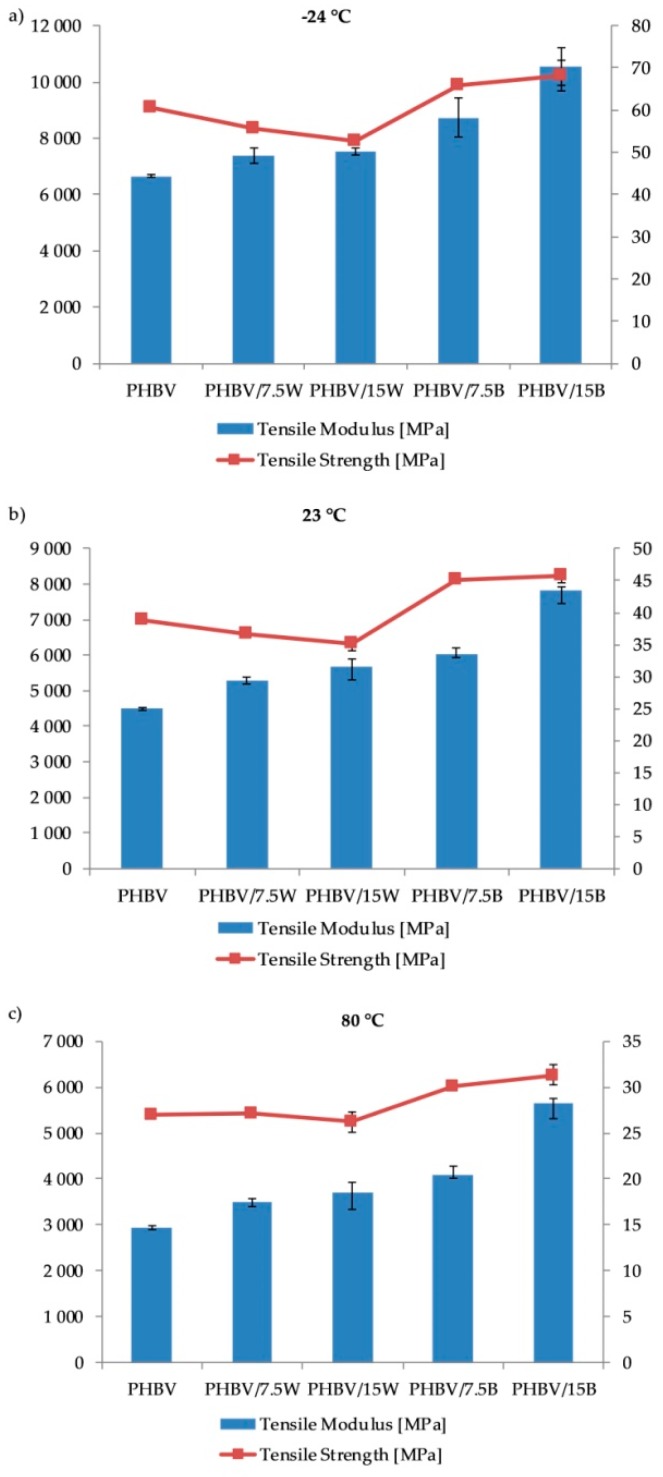
Tensile properties of PHBV and its composites at −24 (**a**), 23 (**b**) and 80 °C (**c**).

Figure 3a–c comprise the results tensile test at various temperatures (−24 °C, +23 ± 2 °C and +80 °C). The addition of WF negatively affected the tensile strength of the tested composites. With increase in the content of WF, tensile strength decreased up to 10% (from 38.7 MPa for neat PHBV to 35.1 MPa at +23 ± 2 °C) for PHBV/15W. This is mainly due to the lack of effective stress transfer between the matrix and the fiber, due to the different nature of the surface of the filler which is hydrophilic and the matrix which is hydrophobic. Generally, tensile strength depends mainly on the matrix and on the interaction and good adhesion between the matrix and fiber. During the stretching of fiber composites, shear stress develops, transferring matrix/fiber loads. Therefore, poor fiber adhesion leads to deterioration of strength. As seen in the SEM images, not all WF were firmly placed in the matrix, causing local deformations to appear along the fiber, and consequently weakening the composite. Besides, it was observed that with the increase of fiber content, the value of the modulus increased. For PHBV/15W, a 26% increase was recorded (from 4500 MPa for PHBV to 5667 MPa at +23 ± 2 °C). As seen in the SEM photographs, the even distribution of fibers in the matrix is responsible for improving the value of Young’s modulus. These results were confirmed by Singh et al. [20] who investigated the mechanical properties of PHBV composites with 10–40% by weight of maple WF content (MWF). The addition of 10% by weight of MWF led to a 6.3% decrease in tensile strength, while the values of Young’s modulus were by 28% higher compared to PHBV. Similar relationships were described by Gunning et al. [25] who examined the mechanical properties of polyhydroxybutyrate composites with plant fibers such as hemp and jute comprising 10–30% by weight. Studies have shown that regardless of the type of natural fibers introduced, tensile strength, and impact strength decreased. However, flexural modulus values increased and the highest values were attained for jute composites due to the higher fiber adhesion to the matrix, which was confirmed by FTIR analysis.

In the case of PHBV/BF, all mechanical parameters improved at all tested temperatures. The highest values were recorded at reduced temperatures, while the lowest values were at elevated temperatures. Addition of fibers by 15% by weight increased Young’s modulus by 74% (+23 ± 2°C) as compared to neat PHBV. The tensile strength of neat PHBV was found to be 4500 ± 39 MPa while it was 7819 ± 106 MPa for PHBV/15B. In the case of tensile strength, the differences between the composites with fibers comprising 7.5% by weight and 15% by weight were insignificant and were 45.0 ± 0.2 MPa and 45.7 ± 1.1 MPa, respectively. The practice of BF being added to polymer matrices is already known [26,27].

**Figure 4:**
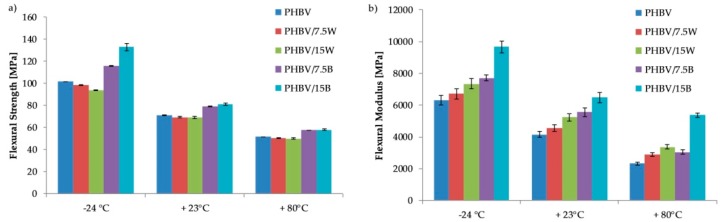
Flexural properties of PHBV and its composites at −24, 23 and 80 °C: (**a**) flexural strength and (**b**) flexural modulus.

Similar relationships, as in the case of tensile strength, have been observed for flexural strength (Figure 4a,b). The addition of WF caused a decrease in flexural strength, and significantly increased the flexural modulus at all temperatures. On the other hand, reinforcement the polymer with BF resulted in improvement of all parameters (flexural strength and flexural modulus) at all temperatures.

The same observations were made by Singh et al. in their study on PHBV composites reinforced with bamboo fiber [28]. Flexural modulus was improved by 100% with 30% fiber by weight and by 154% with 40% fiber by weight, whereas flexural strength remained at the same level of about 30 MPa. The authors pointed out that this fact had been achieved by a reasonable amount of compatibility and interphase interaction between the cellulose fiber and the natural polyester.

It is worth noting that flexural strength values are about 50% higher than tensile strength in all cases. This effect is related to the addition of fibers to the matrix in an almost perpendicular direction to the bending force, as confirmed by SEM images. This arrangement, therefore, results in more resistance to bending than stretching where the force acts in parallel. In addition, stresses occurring during the tensile test appear throughout the cross-section, whereas during bending test they occur in the upper layers where the probability of defects leading to crack initiation is lower.

### 2.4. Micromechanical Analysis

**Figure 5:**
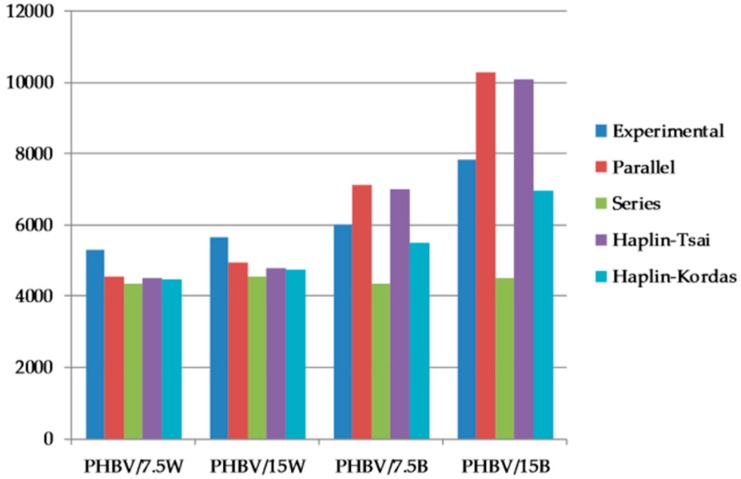
Comparison of experimental results with Parallel, Series, Haplin-Tsai and Haplin-Kordas micromechanical models for the Young’s modulus of composites.

In order to confirm and thoroughly explain the mechanisms of strengthening WF and BF, the experimental data were compared with theoretical values and were plotted in Figure 5. For calculations, models very often used for mechanical modeling of injected composites were used: Ressus, Series, Haplin-Tsai, and Haplin Kordas. The closest values for the theoretical and experimental results were obtained for wood composites, but the experimental values were higher than those obtained from any of the presented models. This may be related to the adopted Young’s modulus and the value of *l/d*. The highest differences in these results were observed for PHBV/BF composites. The highest values were recorded for the Parallel model which assumes a 0° position. However, in the SEM images, it can be observed that not all BFs are arranged in this way. Also, higher values were observed for the HT model, which assumes a perfect connection between phases and, additionally, aligning of all particles in one direction. But in the SEM pictures a random arrangement of fibers could be observed. That is why the most similar results were obtained for the HK model which assumes that the fibers are randomly distributed in the matrix. These models show that after optimizing the adhesion and distribution of fibers, the results of the tested composites may result in even higher improvement in properties than those obtained in the presented studies.

Similar relationships were obtained by Jonoobi et al. [29] who studied mechanical properties of cellulose nanofiber introduced to the PLA matrix. They compared the experimental results to two micromechanical models: Halpin-Tsai and Krenchel. As the fiber content increased, the values obtained during the tensile tests became significantly lower than those determined by calculations. Experimental values are most similar to the Krenchel model. As the authors themselves emphasize, this fact results from randomly distributed fibers.

### 2.5. Hydrothermal Aging

**Figure 6:**
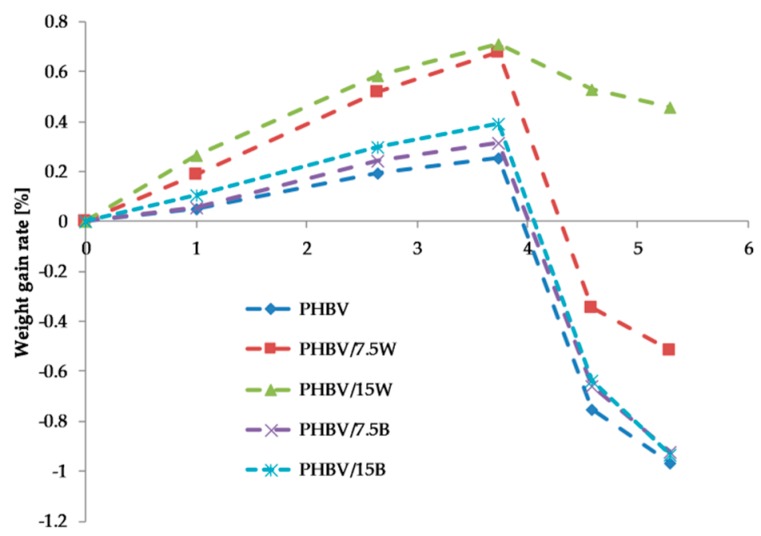
The weight gain rate (M_t_) in function of the square root of the time.

Figure 6 presents the weight-gain rate percentage as a function of the square root of time for PHBV composites with WF and BF in saline solution at 40 °C. Factors that affect the speed of water absorption are: crystallinity of composites, quantity of polar group, crosslink density, structure of fiber, temperature and humidity of environment, microchannels along the filler-matrix. Whereas, the mechanisms of water absorption are: through microcracks, and interfacial gaps that exist due to poor fiber wet ability, capillary transport, sorption by potential gradient induced by the profusion of hydroxyls in the lignocelluloses [30].

The highest water sorption was observed in the first phase of aging, where a linear relationship between (M_t_) and t^1/2^ was visible. In this case, the water diffused from high concentration (water) to low (composite). This behavior is consistent with Fick’s law. By the 14th day of aging, the mass of the composites increased, while after the saturation of the composites with water, there was a decrease in mass. This is most likely related to the degradation of the material due to the breaking of polymer chains. The highest water sorption was found in composites with lignocellulosic fillers (PHBV/15W: M_m_ of 0.71%; PHBV/7.5W: M_m_ of 0.68%). This is related to the hydrophilic nature of WF. The increase in fillers for both WF and BF increased water sorption. The lowest water sorption was recorded for neat PHBV (M_m_: 0.25%). Although BFs are materials that do not absorb water, as shown by SEM photos they create empty spaces between the matrix and fiber which create micro-channels accelerating the absorption of water. Therefore, higher water sorption was recorded for PHBV/15B than PHBV/7.5B, due to the increased number of micro-channels and cracks in the former.

Table 2 presents the summarized values of maximum moisture content and diffusion coefficient. The lowest values for both M_m_ and *D* were observed in the neat polymer. It may be noted that these two parameters were significantly influenced by the type of additive as well as its quantity. With increase in both fibers, the maximum moisture content and the diffusion coefficient increased, but the highest results were obtained for lignocellulose fillers. It is related to the hydrophilic nature of WF and the not too strong interaction between the matrix and fiber which creates an additional lumen of fiber. The addition of 7.5% and 15%by weight of WF resulted in more than twice the increase in the diffusion coefficients compared to BF with the same amount of fibers. Similar results were obtained by Jiang et al. [31]. They performed hydrothermal aging on injection molded PLA-based composites with short jute fiber at 23, 37.8, and 60 °C. The fastest water absorption occurred in the first few days. Of significance was the temperature at which biodegradation took place. The aging process was the fastest at 60 °C.

**Figure 7:**
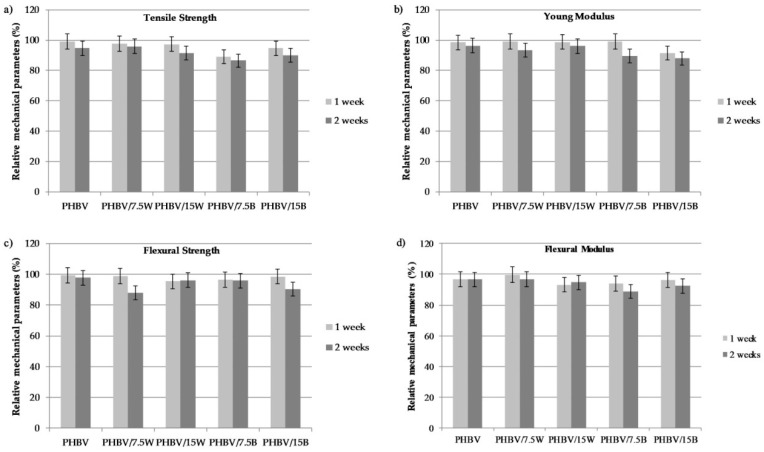
Relative percentage values of strength and modulus versus the initial composites for biodegraded samples.

Figure 7 presents the relative percentage values of strength and modulus of the initial composites with respect to the biodegraded samples (initial parameter/parameter after first week of degradation and after second week of degradation). A slight decrease in mechanical properties was observed after the first week of biodegradation. More decrease in mechanical values occurred after two weeks of aging. As the SEM photographs showed, BF was characterized by poorer adhesion and this was also confirmed by the results of the mechanical tests after biodegradation. More decrease was observed in the values for BF composites after two weeks; this is associated with penetration by water which entered the composite and increased the ability of the fiber to detach from the matrix. Compared to neat PHBV, the decrease in Young’s modulus after two weeks was 9.8% and 3.4% respectively for 7.5% and 15% by weight of BF and 5.9% and 2.7% respectively for 7.5% and 15% by weight of WF. Similar results were obtained by Ventura et al. [32]. They studied biocomposites based on a PHA reinforced with flax fibers. The stiffness of the composites decreased after 100h of incubation in water at 23 °C and 65 °C. The higher the temperature, the lower the recorded values were.

## 3. Materials and Methods

### 3.1. Materials

PHBV grade PHI002 used was manufactured by Nature Plast (Isf, France). This polymer has the following properties: density: 1.25 g/cm^3^; fluidity index (190 °C/1.16 kg): 15–30 g/10 min.; tensile modulus: 4 200 MPa; un-notched Charpy impact strength: 5 kJ/m^2^; and thermal resistance (HDT B): 134 °C.

WF (Lignocel C120) used were manufactured by J. Rettenmaier&Söhne GmbH, (Rosenberg, Germany). The particles size was 70–150 µm.

Chopped BF (BCS17-6.4-KV16) were provided by Basaltex Inc. (Wevelgem, Belgium).Their diameter was 17 μm, length 6.35 mm, density 2.67 g/cm^3^, tensile modulus 84 GPa, and tensile strength 1 180 MPa.

#### Preparations of Biocomposites

PHBV/BF and PHBV/WF biocomposites were obtained using an injection molding equipment, Engel ES 200/40 HSL (Schwertberg, Austria). Injection molding was carried out in the laboratory of plastics technology operating under Grupa Azoty SA (Tarnów, Poland). The size of the samples was 10 × 4 × 150 mm (dumb-bell shape). The samples were described as follows: PHBV/7.5W: 7.5% by weight of WF; PHBV/15W: 15% by weight of WF; PHBV/7.5B: 7.5% by weight of BF and PHBV/15B: 15% by weight of BF. The composite materials were manufactured using a co-rotating twin-screw extruder Maris America Corp., (Windsor Mill, MD, USA). The following parameters were set during the injection process: feed throat: 21 °C; temperatures in individual zones: 170 °C, 165 °C, and 160 °C; mold temperature: 20–25 °C; screw speed: 50 rpm and back pressure: >85 bar. The same temperatures were set for all composites to achieve low melt flow and avoid thermal degradation.

### 3.2. Methods of Testing

#### 3.2.1. Fractographic Observations

Scanning electron microscope (SEM) analysis was done to describe the nature of the failure and explain the damage morphology by employing a low vacuum microscope, JEOL 5510LV (Tokyo, Japan). The examined samples that fractured during the tensile test were coated with a thin gold layer using vacuum coater Cressington (Watford, UK) before observation.

#### 3.2.2. Coefficient of Thermal Expansion

Thermal analysis was carried out on a NETZSCH 402 F1 Hyperion device (Selb, Germany) with Proteus software (version 5.2). Measurements were performed between −30 °C and 130 °C with a heating/cooling rate of 10 °C/min. Samples were placed vertically. The coefficient of thermal expansion was calculated on the basis of the results using the following formula (1):

(1) $$\propto_{L}\;=\;\frac{1}{L}\frac{dL}{dT},\;1/K$$

where $\propto_{L}$ is initial dimension of the sample and dL and dT are the first derivatives of the dimension and temperature, respectively.

#### 3.2.3. Tensile, Bending and Impact Test

The tensile and three-point bending tests were performed using a universal MTS Criterion Model 43 testing machine (Eden Prairie, MN, USA). The tensile test was performed with a constant cross-head speed of 5 mm/min, and the elongation was determined using the MTS axial extensometer and cross-head displacement. The three-point bending test at the cross-head rate of 5 mm/min (up to 5% strain) was performed in compliance with the PN-EN ISO 178 standard. Furthermore, the tests were run at room temperature (+23 ± 2 °C), and at decreased (−24 °C) and elevated (+80 °C) temperatures which reflect the lowest and highest temperatures at which PHBV composites can find practical applications. Before the tests, the samples were kept in a Samsung AZ8 refrigerator (for −24 °C) and an Instron thermal chamber (+80 °C), and conditioned at the test temperatures for 60 min.

The reported average data were based on the test results of at least five standardized samples, along with calculated standard deviations to evaluate test reproducibility.

The un-notched Charpy impact strength (a_cU_) was measured using a Zwick/Roell MTS-SP (Ulm, Germany) testing machine with a 1.5 J hammer.

#### 3.2.4. Micromechanical Models

To examine the characteristics of polymer composites, it is important to compare the experimental values and theoretical results obtained from micromechanical models. In the case of short fiber-reinforced thermoplastics, Young’s modulus can be experimentally determined or estimated. Some theoretical models that are used for the mathematical prediction of mechanical properties of composites are presented below:

Parallel or Voigt:

The two basic models representing the upper and the lower bounds for many properties of composites are the Rule of Mixtures and the so-called Series model. In the Rule of Mixtures model, also referred to as the Parallel model, each phase is assumed to contribute independently to the overall modulus in proportion to its volume fraction. This model assumes an ideal adhesion of the filler to the matrix, which results in overestimating the properties. The following formula is applied to calculate the values as per the Parallel model (2) [33].

(2) $$E_{c}\;={\;E}_{f}*V_{f}+E_{m}+V_{m}$$

where E*_c_*, E*_f_*, and E*_m_* stand for the elastic modulus of composites, fibers, and matrix, respectively, V*_m_* is the volume of matrix and V*_f_* is the volume of friction of fibers described as (3):

(3) $$V_{f}\;=\;\frac{f}{f+\left(1-f\right)\frac{\rho_{f}}{\rho_{m}}}$$

Series or Reuss model:

This model inverts the Rule of Mixtures and assumes no contact between particles. Thus the contribution of particles is confined to the region of the matrix embedding them (4) [34].

(4) $$E_{c}={\left(\frac{1-V_{f}}{E_{m}}+\frac{V_{f}}{E_{f}}\right)}^{-1}$$

Halpin-Tsai (H-T) model:

The H-T model is used to predict the stiffness of the unidirectional composite as a function of filler loading and aspect ratio. This theory assumes that single fibers encased in a cylindrical shell of the matrix embedded in an infinite medium have the average properties of the composite (5) [35].

(5) $$E_{c}=\left(\frac{1+{\mathrm{ABV}}_{f}}{1-{\mathrm{BV}}_{f}}\right)E_{m}$$

The constants *A* and *B* are defined by the following expression (6) and (7):

(6) A=2l/d

(7) $$B=\left(\frac{E_{f}}{E_{m}}-1\right)/\left(\frac{E_{f}}{E_{m}}+A\right)$$

where *l* and *d* refer to the length and diameter, respectively, of the fiber after processing.

Halpin-Kardos (HK) model:

HK model is applied to composites reinforced with fibers randomly distributed in the matrix. It takes into account the properties of the matrix and reinforcing additives along with their proportions and geometries (8) [17].

(8) $$E_{c}=\left\{\frac{3}{8}\left[\frac{1+2{\left(L/D\right)}_{F}H_{L}V_{f}}{1-H_{L}V_{f}}\right]+\frac{5}{8}\left[\frac{1+2H_{T}V_{f}}{1-H_{T}V_{f}}\right]\right\}$$

In this case, *H_L_* and *H_T_* are calculated by the following Equations (9) and (10):

(9) $$H_{L}=\left(\frac{E_{f}}{E_{m}}-1\right)/\left(\frac{E_{f}}{E_{m}}+2\frac{L}{D}\right)$$

(10) $$H_{T}=\left(\frac{E_{f}}{E_{m}}-1\right)/\left(\frac{E_{f}}{E_{m}}+2\right)$$

The diameters and lengths of the WF and BF were determined from the SEM images. The averaged parameters, after processing for all components used in each calculation, are provided in Table 3. All these values were used to predict Young’s modulus of PHBV composites.

#### 3.2.5. Hydrothermal Aging

The standardized samples were subjected to hydrothermal aging. They were immersed in a saline solution (2% by weight salt) at 40 °C to simulate the human body environment. The samples were removed from the tank after 1, 7, 21, and 28 days. The water remaining on the surface was wiped with a tissue paper and the samples weighed. Finally, water uptake was calculated as the mass difference, expressed in percentage using the following formula (11):

(11) $$M_{t}=\left[\frac{W_{t}-W_{0\;}}{W_{0\;}}\right]\times 100,\;$$

where *M_t_* stands for the percentage of water content, *W_t_* for the instantaneous weight of the sample, and *W_0_* for the initial weight of the sample.

Additionally, the tensile test was performed after 7 and 14 days of hydrothermal aging. Because the plot of the weight-gain rate *(M_t_)* has a linear character in the first stage, it can be stated that the absorbed water was controlled by diffusion. In this case, Fick’s second law can be applied according to which the diffusion coefficient *(D)* can be described by the following equation (12) [36]:

(12) $$D_{x}={\left[\frac{M_{1}-M_{2}}{\sqrt{t_{2}}-\sqrt{t_{1}}}\right]}^{2}\times \pi {\left[\frac{h}{4M_{m}}\right]}^{2}$$

where *M_1_* stands for initial moisture content (=0 for completely dry materials), *M_2_* is gained moisture content, *M_M_* is maximum moisture content, *t_1_* is initial time (0 s), *t_2_* is the time when *M_2_* was measured, and *h* is the thickness of the sample.

## 4. Conclusions

PHBV is biodegradable and biocompatible plastic produced naturally by bacteria. It can be a good alternative for many non-biodegradable synthetic polymers and also may be used as an interesting polymer matrix. In addition, PHBV-based composites are becoming competitive to PLA composites, mainly at elevated temperatures. The results from presented study showed that two types of natural fibers gave different reinforcement effect, and the following conclusion were drawn:

(1) Addition of BF increased all tested properties, regardless of the test temperature. The improvement at +24 °C for tensile strength was 18%, modulus of elasticity 74%, impact strength 40%, flexural strength was 30% and flexural modulus over 50%. In the case of elevated temperatures, the highest differences were obtained between neat PHBV and BF composites, where the increase for Young modulus was about 100%.
(2) The addition of WFs results in a quantity proportional to the increase in modulus of elasticity with a slight approximately 10% decrease in strength.
(3) The addition of the fibers to PHBV effectively improved the thermal stability, as the fibre content increased, the values of CTE decreased. The highest over 40% decrease was recorded for composites with 15 wt% of BF.
(4) Additionally, the addition of fibers resulted in an increase in water absorption compared to neat polymer. The water absorption increased for the highest degree of saturation from 0.25% (PHBV) to 0.71% (PHBV/15W). Composites with WF had a higher tendency to absorb water and together with the content of fibers increased the capacity of water uptake, regardless of the type of fibre. In the case of non-absorbable BF, this was poor fiber/matrix adhesion. The water diffusion, with a Fick`s law behavior for the composites, increased with the content of fibers and it`s the highest for composites with 15 wt% of WF –4.68 × 10^−18^ m^2^/s.
(5) After two weeks of biodegradation in saline at 40 °C, a slight decrease of about 10% in strength properties of the tested materials was observed. These studies confirm that biodegradable PHBV-based composites filled with natural fillers can be successfully used as a material for long-life products.
(6) Regarding the mechanical properties, all biocomposites are characterized by higher stiffness that unmodified the polymer. Further work will concern the increase of adhesion of the fibers to the matrix, which will significantly improve the test results.

## Figures and Tables

**Table 1:** Coefficient of thermal expansion.

Sample	Coefficient of Thermal Expansion
PHBV	72.473 × 10^−6^/°C
PHBV/7.5W	59.923 × 10^−6^/°C
PHBV/15W	53.513 × 10^−6^/°C
PHBV/7.5B	48.533 × 10^−6^/°C
PHBV/15B	42.343 × 10^−6^/°C

**Table 2:** Fickan’s diffusion coefficients of poly (3-hydroxybutyrate-co-3-hydroxyvalerate) (PHBV) composites.

Composites	h [m]	M_m_ [%]	D_x_ [×10^−18^ m^2^/s]
PHBV	0.004	0.25	1.26
PHBV/7.5W	0.004	0.68	3.03
PHBV/15W	0.004	0.71	4.69
PHBV/7.5B	0.004	0.32	1.76
PHBV/15B	0.004	0.39	2.06

**Table 3:** Characteristics of components after processing**.**

Components	Length *(l)* [mm]	Diameter *(d)* [μm]	Density [g/cm^3^]	Young Modulus (E) [GPa]
Matrix	-	-	1.25	4.2
Wood fibre	0.3	150	1.52	10
Basalt fibre	4	17	2.67	84
